# High Implementation Adherence to Lenalidomide in Multiple Myeloma

**DOI:** 10.3390/cancers17213587

**Published:** 2025-11-06

**Authors:** Irina Amitai, Hila Magen, Avi Leader, Antoine Pironet, Eric Tousset, Alon Rozental, Sabina De Geest, Iuliana Vaxman, Pia Raanani, Arnon Nagler

**Affiliations:** 1 Division of Hematology, Chaim Sheba Medical Center, Ramat-Gan 5262000, Israel; irina.amitai@sheba.health.gov.il (I.A.); hila.magen@sheba.health.gov.il (H.M.); 2 The Gray Faculty of Medical and Health Sciences, Tel Aviv University, Tel Aviv 6997801, Israel; leadera@mskcc.org (A.L.); alon.rozental@clalit.org.il (A.R.); juliava1@clalit.org.il (I.V.); praanani@012.net.il (P.R.); 3 Institute of Hematology, Davidoff Cancer Center, Rabin Medical Center, Petah Tikva 6356710, Israel; 4 Department of Medicine, Hematology Service, Memorial Sloan Kettering Cancer Center, New York, NY 10065, USA; 5 AARDEX Group, 4432 Ans, Belgium; antoine.pironet@aardexgroup.com (A.P.); eric.tousset@aardexgroup.com (E.T.); 6 Institute of Nursing Science, Department Public Health, University of Basel, 4056 Basel, Switzerland; sabina.degeest@unibas.ch; 7 Academic Centre of Nursing and Midwifery, Department Public Health and Primary Care, 3000 Leuven, Belgium

**Keywords:** adherence, lenalidomide, multiple myeloma, oral anti cancer medications, compliance

## Abstract

Background and Purpose: Adherence to oral anticancer therapy correlates with outcome. Lenalidomide (LEN) is an oral mainstay treatment for multiple myeloma (MM), administered in 21-day/7-day (on/off) cycles. Data on LEN adherence is limited. Electronic monitoring (EM) represents the most reliable adherence assessment method. Experimental Approach: We conducted a prospective observational study using electronic medication event monitoring (MEMS^®^) in lenalidomide-naïve multiple myeloma patients to quantify adherence during on/off cycles and identify patterns of non-adherence in real-world practice. On and off cycles were determined semi-automatically. Implementation adherence was calculated as the proportion of prescribed drug taken, during each on cycle and across all on cycles. Daily adherence predictors were analyzed using logistic regression with generalized estimating equations. Key Results: Eighty-five patients were included. Median age was 68 years, 66% received LEN as a second-line treatment, 75% of patients perfectly adhered to the recommended 21/7-day on/off cycle. Median implementation adherence was 100%. Only 4% of patients had a proportion of doses taken below 90%. All doses were taken by 51% of patients, while 9% missed ≥4 doses. Among the 13 predictors investigated, only age under 80 and participation in a support group were statistically significant. Conclusions: this novel assessment of LEN adherence in MM patients demonstrated high implementation adherence and cycle duration compliance.

## Simple Summary

Taking oral cancer medicines exactly as prescribed is important because it directly affects how well patients do. Lenalidomide is a common oral treatment for people with multiple myeloma, given in cycles of three weeks on the drug and one week off. However, little is known about how closely patients follow this schedule in real life. In this study, we used electronic devices that record when each pill is taken to carefully track how patients used lenalidomide over time. We found that most patients followed their treatment plan very well, with many taking all of their prescribed doses correctly. Older age and lack of support were linked to lower adherence. These findings suggest that most patients are able to take lenalidomide as prescribed, but extra attention may be helpful for older patients or those without support. This information can guide doctors in improving treatment outcomes.

## 1. Introduction

Oral anticancer agents have been a major step forward in hematology and oncology. However, adherence of patients to oral drugs is a major issue, contributing to high interindividual variability of the patients’ drug exposure and suboptimal response [1,2,3,4,5].

Medication adherence is defined as the extent to which an individual’s medication intake behavior corresponds to that agreed with the health professional [6]. In oncology, there is evidence of oral anticancer drugs adherence rates being as low as 46% [3]. Non-adherence to oral anticancer affects therapy efficacy. For example, over a decade ago, it was shown that inadequate adherence to imatinib (namely, ≤90% of prescribed dose taken) among chronic myeloid leukemia patients, was associated with suboptimal molecular response [2].

Adherence and persistence to medications have been defined in several ways [7]. In this study, the ABC taxonomy, according to Vrijens et al., was followed [8]. Medication adherence includes three quantifiable phases: (A) initiation (when the patient takes the first dose of a prescribed medication), (B) implementation (the extent to which a patient’s actual dosing corresponds to the prescribed dosing regimen, from initiation until the last dose), and (C) discontinuation (when the patient stops taking the prescribed medication, against medical advice).

Multiple myeloma (MM) accounts for approximately 10% of hematologic malignancies [9] and predominantly affects the elderly population, with a median age at diagnosis of 69 years (SEER data) [10,11,12]. Over the past fifteen years, MM survival outcomes have improved dramatically, initially driven by the introduction of bortezomib, thalidomide, and lenalidomide (LEN) [9]. Subsequently, the therapeutic armamentarium has expanded to include more novel agents: carfilzomib, pomalidomide, ixazomib, elotuzumab, daratumumab, isatuximab, selinexor, belantamab mafodotin, chimeric antigen receptor T (CAR-T) cell therapies, and lately the bispecific T-cell engagers (BiTEs), i.e., teclistamab, talquetamab and elranatamab [9]. Contemporary treatment protocols frequently incorporate at least one oral agent, offering patients unprecedented convenience [13]. The evolving standard of care for newly diagnosed MM patients, both eligible and non-eligible for autologous transplant, consists of a quadruplet regimen, including bortezomib, LEN, anti CD38 immunotherapy and a corticosteroid [13,14,15]. Maintenance with LEN is incorporated in most regimens, highlighting the role of LEN in patient care and hence, the importance of prolonged adherence to therapy. Indeed, the shift towards oral administration raises concerns regarding potential non-adherence, particularly among elderly patients who often contend with polypharmacy and may struggle with the added responsibility of maintaining their own healthcare. Moreover, elderly and/or frail patients frequently prove ineligible for more intensive therapeutic approaches such as CAR-T cell therapy, as they are more prone to infections and other complications, rendering their treatment regimens even more dependent upon oral agents [16]. Importantly, previous studies have shown that lower life expectancy and declining functional status are often associated with reduced medication adherence in elderly patients, further emphasizing the need for structured adherence support in this population.

LEN is an oral mainstay of modern MM treatment, usually given in 21 day/7 day or 14 day/7 day (on/off) cycles [17]. Therefore, adherence to LEN in MM entails both adherence to duration of on/off cycles as well as implementation adherence (i.e., dosing/timing issues). Data on adherence to LEN in MM is limited and variable. Adherence assessment is mostly limited to retrospective pharmacy refill records, chart reviews and self-reported patient questionnaires [18,19,20,21], and there is no data on adherence to the duration of LEN on/off cycles.

In fact, a recent systematic review and meta-analysis on adherence to oral agents in MM included 19 observational studies, and the calculated overall pooled proportion of adherent patients was 68%. Quite expectedly, adherence was higher in self-reported questionnaire-based studies compared to those using prescription/dispensing data [22]. Measuring adherence might be challenging, which accounts for varying estimates. Electronic monitoring (EM) has emerged as an accurate way to capture the dynamic and longitudinal characteristics of drug intake during the implementation phase of adherence [23].

Using EM allows the assessment of both daily implementation adherence and duration of on/off cycles [24].

Thus, the aim of our study was to assess adherence to the duration of LEN on/off cycles and patterns of EM implementation adherence to LEN during on cycles in a real-world setting. Second, the prevalence of several types of non-adherence was studied. Third, the link between adherence and socio-demographic and clinical variables was also studied. To our knowledge, this is the first prospective study to evaluate adherence to lenalidomide using electronic monitoring, capturing both daily implementation and on/off cycle duration in multiple myeloma patients.

## 2. Methods

This was a prospective observational study including a convenience sample of LEN-naïve adult patients receiving once-daily LEN-based regimens for the treatment of MM at two tertiary academic medical centers in Israel.

All consecutive patients that were starting treatment with LEN were approached and offered to participate in the study. In Israel, the public healthcare system provides universal health insurance with full reimbursement of approved anticancer drugs, ensuring equal access to lenalidomide therapy regardless of socioeconomic status.

The study was registered with clinicaltrials.gov (NCT02733224) and approved by the local institutional review boards.

The eligibility for this study included patients receiving LEN for MM. Patients were excluded if life expectancy was <6 months, anticipated duration of LEN therapy was <3 months, or they did not self-administer medications. Medication adherence and age were not part of the eligibility criteria. All participants provided written informed consent.

Several socio-demographic and clinical variables were collected at baseline.

The patients arrived to the clinic on a monthly basis or more often. The refills were performed during their regular follow-up visit. As this was a non-interventional observational study, blinding was not applicable. Adherence data were automatically recorded by MEMS^®^, and study staff (clinical coordinators) were not involved in data analysis, minimizing potential bias.

### 2.1. Variables and Measurements

Several patient characteristics were measured at baseline: patient demographics, disease characteristics, information about the medication regimen, etc. The complete list is provided in Supplementary Table S1.

### 2.2. Adherence Measurements

Medication adherence during implementation was monitored using EM (MEMS^®^, AARDEX Group, Seraing, Belgium) for a maximum 18 months. LEN was stored in bottles closed with an electronic cap, the MEMS^®^ Cap, which recorded the date and time of each bottle opening as a proxy for LEN intake. Patients gave informed consent and were told about the EM adherence assessment. Periods during which treatment was interrupted following doctors’ instructions or during which EM data was unavailable for technical reasons were documented as “non-monitored periods” and censored from the analysis.

Because LEN is taken in a succession of 21-day on/7-day off cycles, the usual methods for computing medication adherence had to be adapted to this specific regimen.

First, the start and end dates of each on/off cycle had to be precisely known. However, logistic considerations can interfere with the planned start and end dates: moved appointments and catching-up for missed doses during an on cycle [25]. Study staff documented the start and end dates of on and off cycles in MEMS^®^ Adherence Software (Version 4.3, AARDEX Group, Seraing, Belgium). When this data was missing, the start and end dates of on cycles were determined semi-automatically: an algorithm for the detection of sudden changes in medication adherence was applied [26] and the results were manually curated by the authors by visual inspection of the results. Off cycles were defined as periods between on cycles.

From this information, implementation adherence during each on cycle was quantified using the proportion of prescribed dose taken, computed as the number of intakes during the on cycle, divided by the number of days in the on cycle. The proportion of prescribed drug taken was also computed across all on cycles for a given participant. Treatment interruptions translate into a proportion of prescribed dose taken below 100%, while overdosing translates into a value over 100%. The number of patients with a proportion of doses taken below 90% was calculated, since this cutoff was clinically relevant in a prior study of chronic myeloid leukemia patients [21]. Adherence to off cycles was computed as the number of patients who took at least one dose during an off cycle. These metrics only relate to the implementation component of adherence and do not take non-initiation or treatment discontinuation into account.

On several occasions, patients were instructed to stop taking LEN during specific periods. Such periods are called “non-monitored periods” and were censored from the analyses.

### 2.3. Statistical Analysis

Classical descriptive statistics were used to describe socio-demographic data at baseline, and clinical data at baseline. Numbers and percentages were adopted to characterize binary and categorical variables; median and 1st and 3rd quartiles were used to summarize quantitative variables.

Descriptive statistics were calculated as appropriate.

The link between implementation adherence and predictors was studied longitudinally. Let Yik be a binary variable indicating whether participant i took at least one dose on day k. More specifically:

- Yik = 1 if day k is during an on period and participant i took at least one dose;
- Yik = 0 if day k is during an on period and participant i took no dose;
- Yik is not defined if day k is during an off period or during a non-monitored period.

As a consequence, doses taken during off periods were not considered in the analysis.

The link between Yik and predictors was investigated using a logistic regression where dependence among observations from a given participant over time was modeled through a generalized estimating equations approach with a first-order autoregressive covariance structure [1,27].

Thirteen covariates (age, education level, number of pills per day, line of therapy, relationship status, participation in a support group, site at which the patient was treated, to account for potential differences between sites, and more, see Supplementary Table S2) were considered to be particularly relevant to adherence and were individually tested for their effect on adherence using logistic regressions adjusted for repeated binary data (generalized estimating equations logistic regression model) [1,27].

Missing values for categorical variables were coded as a separate category. Missing values for continuous variables (age, disease duration, and number of pills per day) occurred in less than 10% of cases and were dropped.

Analysis of residuals was performed to investigate whether nonlinear models (quadratic or cubic) should be tested. If quadratic or cubic effects were found to be significant, the analysis for the variable of interest was repeated by categorizing the variable. The categories were chosen to best fit the nonlinear effects.

The significance level was taken at 0.05/13 = 0.0038, where the division by 13 is a Bonferroni correction to account for multiple testing [2,28]. Models were fitted using Proc GENMOD in SAS software, version 9.4 of the SAS System for Windows.

## 3. Results

Patients were enrolled between May 2016 and November 2019. Ninety-three patients provided written informed consent. Eight were excluded due to inability to initiate use of the EM device and no patients were lost to follow-up. Eighty-five patients were included in the analysis. Complete baseline demographic and clinical characteristics are shown in Supplementary Table S1: 27 (32%) females; median age 68 years [Q1–Q3: 60–74]; median no. of pills/day: 5 [Q1–Q3: 4–7]; median prior disease duration: 19 months [Q1-Q3 5.75–45.7]; regimen: LEN-dexamethasone with (n = 43, 51%) or without (n = 38, 44%) additional drugs (5% missing); treatment line: 1st = 15%, 2nd = 66%, and 3rd = 15% (4% missing). The median duration of follow-up was 133 days [Q1–Q3 77–152] and the median number of cycles was 3 [IQR 1–4].

**Figure 1:**
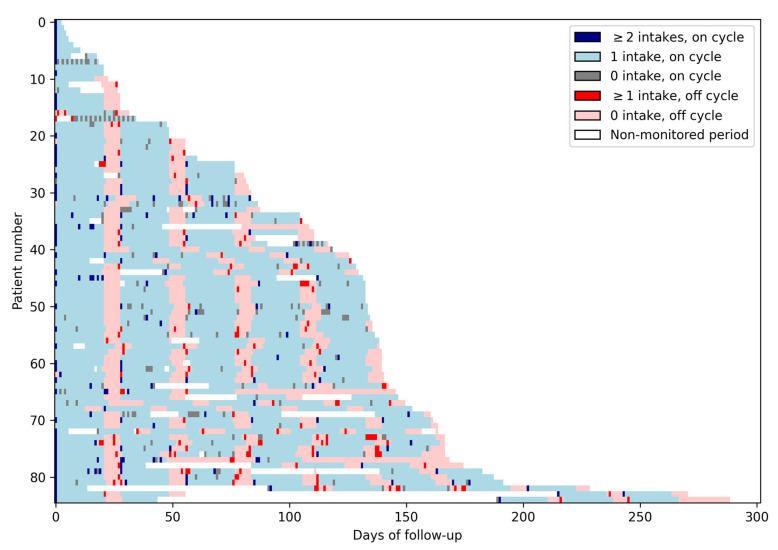
Lasagna plot. This figure presents the medication intake data of the whole study population. Each row corresponds to one patient, and each column corresponds to a day. Each light blue cell represents an on day with one intake; each dark blue cell represents an on day with two intakes or more; each gray cell represents an on day with no intake. Light red cells represent off days with no intakes; bright red cells represent off days with at least one intake, and each white cell represents a non-monitored day. The alternance of on and off cycles is visible in this Figure as the alternance of blue and red areas. Missed doses during on cycles correspond to gray cells, while doses taken during off cycles correspond to bright red cells.

Figure 1 presents the data collected daily for each of the 85 patients. The alternance of on and off cycles is visible in this Figure as the alternance of blue and red areas. Missed doses during on cycles correspond to gray cells, while doses taken during off cycles correspond to bright red cells.

The median duration of on/off cycles was 21 [Q1–Q3: 21–21] and 7 [Q1–Q3: 7–7] days, respectively (Figure 1). Perfect adherence to on/off cycle (21/7) duration occurred in 75% of patients (64/83). Twenty-four patients had at least one additional off day between cycles and 7 had at least one off cycle shorter than 7 days.

**Figure 2:**
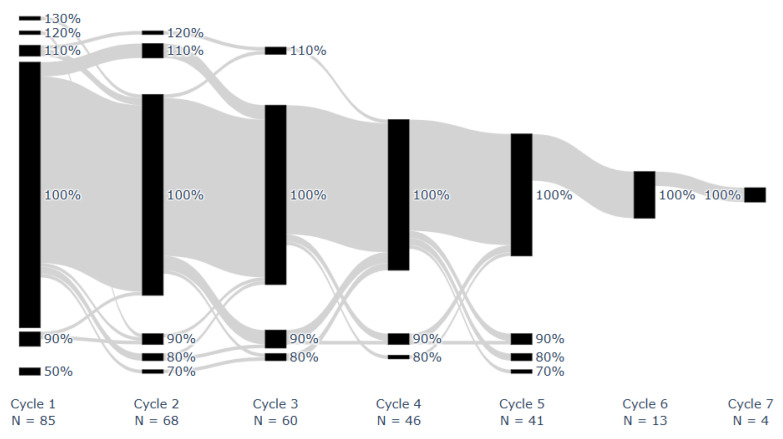
Proportion of prescribed dose taken for each patient during each on cycle. Successive cycles of individual patients are connected using gray lines. Adherence was rounded to the nearest ten.

Median overall LEN implementation adherence was 100% [IQR: 98–100%; range: 50–113%]. Median LEN implementation adherence per cycle was 100% [IQR: 100–100%; range: 50%, 130%]. Figure 2 shows the proportion of doses taken for each patient during each monitored on cycle. All doses were taken by 43 patients (51%). One missed dose occurred in 18 (21%) patients, two in 9 (11%), three in 7 (8%), and 8 (9%) had ≥4 missed doses, at any stage. Two or more consecutive doses were missed once in 8 patients (9%) and twice in 2 (2%).

Fifty-two took at least one dose during their scheduled off week. Four percent of patients demonstrated implementation adherence below 90%.

**Figure 3:**
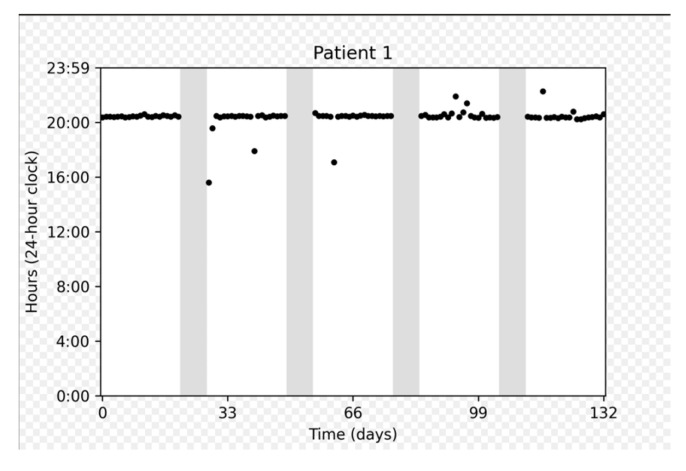

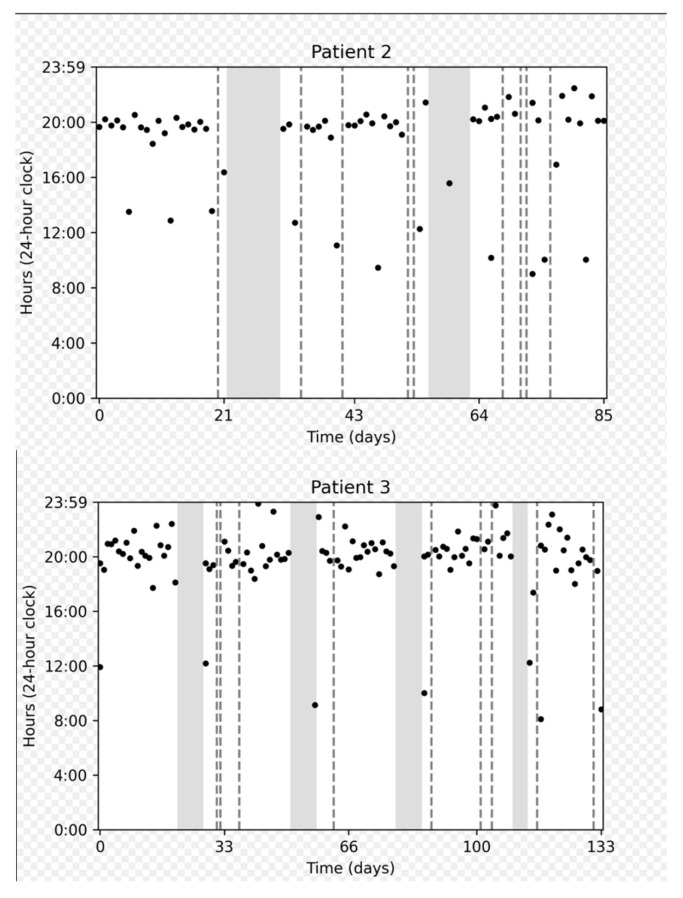
Chronological plots representing individual data from three patients. The horizontal axis displays the intake date. The vertical axis gives the time on a 24 h clock from 0:00 to 23:59. Each dot represents one medication intake. The vertical dashed gray lines represent days with no intake. Gray areas represent “off” periods, and white areas represent “on” periods.

Figure 3 depicts the adherence data collected for several representative participants: a participant who demonstrated perfect adherence a participant with a longer on cycle to compensate for a missed dose, and a participant who experienced multiple interruptions.

On cycles shorter than 21 days were associated with a number of doses taken approximately equal to the duration of the cycle (e.g., duration = 20 days, number of doses = 19). On the other hand, on cycles longer than 21 days approximately corresponded to 21 doses taken, meaning that patients prolonged the on cycle to make up for missed doses.

The following two predictors were found to be significantly associated with better adherence: age below 80 years (*p* = 0.0012) and participation in a myeloma patient support group (*p* = 0.0028). The complete results of the univariate models linking clinical and socio-demographic predictors to longitudinal adherence are presented in Supplementary Table S2.

## 4. Discussion

This was a prospective observational study including a convenience sample of LEN-naïve adult patients receiving LEN-based regimens for once-daily treatment of MM in Israel. Because LEN is taken in a succession of 21-day on/7-day off cycles, a new methodology was developed to compute adherence to such a regimen.

Our results demonstrate that perfect adherence to the recommended 21-day on/7-day off cycle duration was achieved in 75% of patients, with deviations primarily reflecting dose adjustments.

We also investigated the socio-demographic and clinical predictors of implementation adherence to an on/off regimen. The two significant predictors were age below 80 years and participation in a myeloma patient support group. Younger patients may demonstrate better cognitive function, routine flexibility, and familiarity with electronic monitoring. The fact that participation in a support group is associated with better adherence can be linked to the healthy adherer effect: patients who adhere better to their medications also tend to follow other recommendations [29].

High implementation adherence rates suggest that most patients are capable of integrating LEN into their daily routines, despite its complex on/off schedule. This is of key importance in this patient population, where oral agents play a major role in therapy regimens. The observed deviations raise questions about the clinical significance of non-adherence to on/off cycles and the impact on treatment efficacy. Of note, most participants were taking several medications for other medical conditions before the start of the study, meaning that they were already used to taking medications. This is a potential contextual factor that might have contributed to high adherence.

In the present study, patients with shorter on cycles adhered to the corresponding number of doses, while those with prolonged on cycles appeared to compensate for missed doses. A similar behavior was observed in a study of adherence to on/off cycles for palbociclib [25]. This behavior underscores the adaptability of patients and the potential for individualized treatment approaches. Despite high adherence rates, 25% of patients deviated from the prescribed on/off schedule, suggesting that even minor non-adherence warrants further exploration regarding clinical outcomes. Importantly, 4% of patients demonstrated implementation adherence below 90%, a threshold associated with suboptimal outcomes in other hematological malignancies such as chronic myeloid leukemia [2].

As oral therapeutics become progressively integrated into oncological practice, improving patients’ adherence assumes critical significance. This is further amplified considering potential impediments to clinic attendance, including declining functional status, difficult transportation to the clinic, or unforeseen circumstances such as the COVID-19 pandemic. Adherence assessment in MM has traditionally relied on retrospective methods, such as medication possession ratios (MPRs) and self-reported questionnaires, which are prone to overestimation and fail to capture nuanced medication-taking behavior. Our findings align with the results of Cransac et al. [18] and Santoleri et al. [19], who reported variability in adherence rates due to methodological differences. EM data, in contrast, offers a unique granularity, enabling the assessment of both adherence to dosing schedules and patterns of missed doses.

EM also provides opportunities for tailored interventions. Personalized feedback based on EM data, termed Electronic Monitoring Feedback (EMF), could empower patients to address non-adherent behavior proactively. This approach has shown promise in improving adherence and clinical outcomes in other settings [25,27,30]. Integration of EMF into routine clinical practice could enhance patient engagement and optimize therapy in MM.

This study’s strength is its real-world setting. However, it is limited by its observational design and the potential for selection bias, as patients who consented to EM may represent a more adherent population. Of note, because drug costs are fully reimbursed within Israel’s public health system, financial barriers to adherence were minimal, allowing assessment of behavioral and clinical rather than economic determinants of adherence. Indeed, the observational design of this study inherently limits causal inference, and potential unmeasured confounders (e.g., variations in concurrent supportive therapies or caregiver involvement) may have influenced adherence patterns. The relatively short follow-up period and modest sample size further restrict the ability to evaluate longitudinal trends or correlate adherence with long-term outcomes. These findings should therefore be interpreted as hypothesis-generating and highlight the need for larger, multicenter, prospective studies. Additionally, some participants had missing social and demographic data, and our sample size and geographic scope were restricted, limiting the generalizability of findings. Another limitation is the fact that the beginning and end of on and off periods were sometimes missing and had to be inferred from the medication intake data. As a consequence, in these cases, deviations from the actual expected cycles were not detected, which probably increased the adherence estimates. Deviations from the expected cycles are frequent in on/off regimens [25], and the data-driven detection of cycles was a practical way of overcoming this issue. Despite these limitations, the robust adherence data generated using electronic monitoring underscores this tool’s value as a reliable standard for adherence assessment during the implementation phase.

In the present work, clinical outcomes were not available, so it was not possible to study the link between outcomes and the proportion of prescribed doses taken, specific non-adherence behaviors, or cycle duration. Future research may investigate the clinical thresholds for LEN adherence, particularly regarding on/off cycle deviations, to guide therapeutic decision-making.

With the continuing transition towards oral medications for multiple myeloma, and given that this is a disease primarily affecting the elderly population who face additional difficulties that may create barriers, oncology care teams are embracing their new roles in patient education, regular communication, and adherence promotion. The patients themselves should recognize their increased responsibility for their own healthcare, which is key to treatment success. Thus, patient engagement in a partnership with the oncology care team is the cornerstone of achieving optimal outcomes with oral treatments. More so, patients with MM, across all age spectrums, report continuous patient-specific mental and physical health burdens, indicating high unmet needs throughout the disease journey [28,31], and even addressing these burdens by the healthcare team would most possibly improve the patients’ well-being and their adherence to treatment.

## 5. Conclusions

In conclusion, this study provides the first detailed electronic assessment of lenalidomide adherence at the cycle level in real-world multiple myeloma care. Electronic monitoring proved accurate and objective, revealing high adherence and consistent cycle compliance. These findings support the use of digital monitoring to identify patients at risk of non-adherence and improve treatment continuity.

This real-world prospective study demonstrates that adherence to lenalidomide in multiple myeloma is generally high when assessed through electronic monitoring, although deviations from the prescribed on/off schedule are not uncommon. Electronic monitoring provides a precise and dynamic assessment of medication-taking behavior, surpassing traditional adherence measures in accuracy and clinical relevance. These findings underscore the importance of structured patient education and ongoing support to sustain adherence over prolonged treatment periods.

Future studies should aim to integrate electronic monitoring data with clinical outcomes to define adherence thresholds of therapeutic significance. Incorporating real-time feedback and digital adherence tools into clinical practice may further enhance adherence, particularly among elderly and frail patients, thereby optimizing treatment efficacy and quality of life.

## Data Availability

The datasets generated and/or analyzed during the current study are not publicly available due to patient confidentiality and institutional data protection policies, but de-identified data may be made available from the corresponding author upon reasonable request.

## Supplementary Materials

The following supporting information can be downloaded at https://www.mdpi.com/article/10.3390/cancers17213587/s1, Supplementary Table S1. Baseline patient characteristics; ASCT: autologous stem cell transplant; ECOG: Eastern Cooperative Oncology Group; MM: multiple myeloma; VAS: visual analogue scale; ISS: international staging system, dLFC: difference in free light chains, VDTPACE refers to a combination chemotherapy regimen. Supplementary Table S2. results of the univariate logistic regressions adjusted for repeated binary data (generalized estimating equations logistic regression model) linking clinical and socio-demographic predictors to longitudinal adherence.
