# Supplementary material for: High Implementation Adherence to Lenalidomide in Multiple Myeloma
Source: Cancers (Basel). 2025 Nov 6;17(21):3587. doi: 10.3390/cancers17213587 (PMC12606733; doi:10.3390/cancers17213587)
Supplement: Supplementary file 1 [file cancers-17-03587-s001.zip › cancers-3927127-supplementary.docx]

**Supplementary Table S1.** Baseline patient characteristics; ASCT: autologous stem cell transplant; ECOG: Eastern Cooperative Oncology Group; MM: multiple myeloma; VAS: visual analogue scale; ISS: international staging system, dLFC: difference in free light chains, VDTPACE refers to a combination chemotherapy regimen.

| **Indication for treatment, n (%)** | |
| --- | --- |
| Active disease | 72 (85 %) |
| Maintenance after ASCT | 13 (15 %) |
| **Age at initiation (years), median (Q1 – Q3)** | 68 (60 – 74) |
| **Gender, n (%)** | |
| Male | 58 (68 %) |
| Female | 27 (32 %) |
| **Relationship status, n (%)** | |
| No relationship | 14 (16 %) |
| In a relationship | 67 (79 %) |
| Missing | 4 (5 %) |
| **Living with a partner, n (%)** | |
| No | 14 (16 %) |
| Yes, with a partner | 67 (79 %) |
| Missing | 4 (5 %) |
| **Education level, n (%)** | |
| Less than high school | 8 (9 %) |
| 12 years | 35 (41 %) |
| Academic | 33 (39 %) |
| Missing | 9 (11 %) |
| **Member of support group, n (%)** | |
| No | 65 (76 %) |
| Yes | 17 (20 %) |
| Missing | 3 (4 %) |
| **Number of patients with MM seen last year by hematologist, n (%)** | |
| Less than 20 | 0 (0 %) |
| More than 20 | 80 (94 %) |
| Missing | 5 (6 %) |
| **Received explanation on adherence, n (%)** | |
| No | 30 (35 %) |
| Yes | 52 (61 %) |
| Missing | 3 (4 %) |
| **Patients with diabetes mellitus, n (%)** | |
| No | 71 (84 %) |
| Yes | 12 (14 %) |
| Missing | 2 (2 %) |
| **Patients with chronic heart failure, n (%)** | |
| No | 83 (98 %) |
| Yes | 0 (0 %) |
| Missing | 2 (2 %) |
| **Patients with ischaemic heart disease, n (%)** | |
| No | 78 (92 %) |
| Yes | 5 (6 %) |
| Missing | 2 (2 %) |
| **Patients with hypertension, n (%)** | |
| No | 54 (64 %) |
| Yes | 29 (34 %) |
| Missing | 2 (2 %) |
| **Patients with psychiatric condition, n (%)** | |
| No | 83 (98 %) |
| Yes | 0 (0 %) |
| Missing | 2 (2 %) |
| **ECOG Performance Status Scale, n (%)** | |
| 0 | 38 (45 %) |
| 1 | 12 (14 %) |
| 2 | 5 (6 %) |
| 3 - 4 | 0 (0 %) |
| Missing | 30 (35 %) |
| **MM-related fractures, n (%)** | |
| No | 45 (53 %) |
| Yes | 7 (8 %) |
| Missing | 33 (39 %) |
| **Pain measured by VAS, median (Q1 – Q3)** | 0.5 (0 – 5) |
| Missing, n (%) | 31 (36 %) |
| **ISS stage at diagnosis, n (%)** | |
| Chronic | 14 (16 %) |
| Accelerated | 16 (19 %) |
| Blastic crisis | 16 (19 %) |
| Missing | 39 (46 %) |
| **Disease duration at study initiation (months), median (Q1 – Q3)** | 19 (5.75 – 45.7) |
| **Extra-medullary disease at diagnosis, n (%)** | |
| No | 48 (56 %) |
| Yes | 12 (14 %) |
| Missing | 25 (29 %) |
| **Extra-medullary disease at study initiation, n (%)** | |
| No | 44 (52 %) |
| Yes | 5 (6 %) |
| Missing | 36 (42 %) |
| **Immunofixation at diagnosis, n (%)** | |
| IgG kappa | 26 (31 %) |
| IgG lambda | 22 (26 %) |
| IgA kappa | 7 (8 %) |
| K light chain | 13 (15 %) |
| L light chain | 5 (6 %) |
| IgD lambda | 1 (1 %) |
| IgA lambda | 4 (5 %) |
| IgG kappa and IgA kappa | 1 (1 %) |
| Missing | 6 (7 %) |
| **Quantitaive immunoglobulin assay at diagnosis (mg/dL), median (Q1 – Q3)** | 2,350 (850 – 3,490) |
| Missing, n (%) | 20 (24 %) |
| **Quantitaive immunoglobulin assay at study initiation (mg/dL), median (Q1 – Q3)** | 1,375 (608 – 2,675) |
| Missing, n (%) | 19 (22 %) |
| **dFLC at diagnosis (mg/dL), median (Q1 – Q3)** | 1.99 (0.07 – 54.31) |
| Missing, n (%) | 18 (21 %) |
| **dFLC at study initiation (mg/dL), median (Q1 – Q3)** | 3.47 (0.18 – 62.99) |
| Missing, n (%) | 17 (20 %) |
| **Plasma cells in bone marrow at diagnosis (%), median (Q1 – Q3)** | 45 (30 – 74) |
| Missing, n (%) | 15 (18 %) |
| **Plasma cells in bone marrow at study initiation (%), median (Q1 – Q3)** | 25 (6.5 – 60) |
| Missing, n (%) | 58 (68 %) |
| **Line of myeloma therapy, n (%)** | |
| 1 | 13 (15 %) |
| 2 | 56 (66 %) |
| 3 | 13 (15 %) |
| Missing | 3 (4 %) |
| **Regimen, n (%)** | |
| Lenalidomide and dexamethasone | 38 (44 %) |
| Carfilzomib, lenalidomide and dexamethasone | 9 (11 %) |
| Ixazomib, lenalidomide and dexamethasone | 2 (2 %) |
| Bortezomib, lenalidomide and dexamethasone | 17 (20 %) |
| Daratumumab, lenalidomide and dexamethasone | 14 (16 %) |
| Elotuzumab, lenalidomide and dexamethasone | 1 (1 %) |
| Missing | 4 (5 %) |
| **Type of previous therapy, (%)** | |
| Bortezomib-based therapy (1) | 50 (59 %) |
| VDTPACE (2) | 6 (7 %) |
| Melphalan-based therapy (3) | 0 (0 %) |
| 1 and 2 | 5 (1 %) |
| 1 and 2 | 8 (9 %) |
| 1, 2 and 3 | 1 (1 %) |
| No prior therapy | 8 (9 %) |
| Missing | 7 (8 %) |
| **Previous ASCT, n (%)** | |
| No | 42 (49 %) |
| Yes | 39 (46 %) |
| Missing | 4 (5 %) |
| **Daily number of pills, median (Q1 – Q3)** | 5 (4 – 7) |
| Missing, n (%) | 18 (21 %) |
| **Total daily lenalidomide dose (mg), n (%)** | |
| 5 | 3 (2 %) |
| 10 | 6 (7 %) |
| 15 | 32 (68 %) |
| 25 | 42 (46 %) |
| Missing | 2 (2 %) |
| **Number of other medications, median (Q1 – Q3)** | 5 (3 – 6.75) |
| Missing | 7 (8 %) |
| **Route for additional medications, n (%)** | |
| Subcutaneous | 41 (48 %) |
| Oral | 40 (47 %) |
| Missing | 4 (5 %) |
| **Site, n (%)** | |
| Rabin | 36 (42 %) |
| Sheba | 49 (58 %) |

**Supplementary Table S2.** results of the univariate logistic regressions adjusted for repeated binary data (generalized estimating equations logistic regression model) linking clinical and socio-demographic predictors to longitudinal adherence.

|  | **Coefficient** | **95% confidence interval** | **p-value** |
| --- | --- | --- | --- |
| Time | -0.00 | [-0.00, 0.00] | 0.919 |
| Gender is male | -0.22 | [-0.58, 0.14] | 0.228 |
| Age at study initiation | -0.02 | [-0.07, 0.03] | 0.486 |
| Age at study initiation is > 80 years | -1.52 | [-2.44, -0.60] | 0.001 |
| Education level |  |  |  |
| Less than high school | 0.65 | [0.03, 1.26] | 0.038 |
| 12 years | 0.10 | [0.38, -0.64] | 0.785 |
| Academic | Reference | - | - |
| Missing | 0.33 | [-0.81, 1.47] | 0.574 |
| Relationship status |  |  |  |
| No relationship | Reference | - | - |
| In a relationship | 0.09 | [-0.93, 1.12] | 0.858 |
| Missing | 0.11 | [-1.31, 1.53] | 0.877 |
| Participation to a myeloma patient support group |  |  |  |
| No | Reference | - | - |
| Yes | 1.12 | [0.38, 1.86] | 0.003 |
| Missing | 0.00 | [-1.06, 1.06] | 0.997 |
| Number of pills per day | -0.02 | [-0.14, 0.11] | 0.777 |
| ISS stage at diagnosis |  |  |  |
| Chronic | Reference | - | - |
| Accelerated | -0.47 | [-1.50, 0.56] | 0.370 |
| Blastic crisis | -0.84 | [-2.08, 0.40] | 0.187 |
| Missing | -0.33 | [-1.35, 0.70] | 0.531 |
| Disease duration at study initiation | -0.00 | [-0.00, 0.00] | 0.707 |
| ECOG performance status scale |  |  |  |
| 0 | Reference | - | - |
| 1 | 0.17 | [-0.75, 1.09] | 0.713 |
| 2 | -0.33 | [-1.82, 1.16] | 0.661 |
| Missing | 0.36 | [-0.37, 1.09] | 0.334 |
| Line of myeloma therapy |  |  |  |
| 1 | Reference | - | - |
| 2 | -0.51 | [-1.16, 0.14] | 0.126 |
| 3 | -0.40 | [-1.36, 0.55] | 0.411 |
| Missing | -0.60 | [-1.71, 0.51] | 0.289 |
| Site is Rabin | 0.66 | [0.04, 1.28] | 0.037 |
| Route for additional medications |  |  |  |
| Subcutaneous | Reference | - | - |
| Oral | -0.59 | [-1.26, 0.08] | 0.085 |
| Missing | -1.56 | [-2.95, 0.03] | 0.055 |
